# MEDIUM-TERM FOLLOW-UP RESULTS WITH LAPAROSCOPIC SLEEVE GASTRECTOMY

**DOI:** 10.1590/S0102-6720201500S100017

**Published:** 2015-12

**Authors:** Almino Cardoso RAMOS, Eduardo Lemos de Souza BASTOS, Manoela Galvão RAMOS, Nestor Tadashi Suguitani BERTIN, Thales Delmondes GALVÃO, Raphael Torres Figueiredo de LUCENA, Josemberg Marins CAMPOS

**Affiliations:** 1 Gastro-Obese-Center - Advanced Center of Gastroenterology, Metabolic and Bariatric Surgery, São Paulo, SP; 2 Federal University of Pernambuco, Recife, PE, Brazil

**Keywords:** Morbid obesity, Gastrectomy, Laparoscopy

## Abstract

**Background:**

: The indications for sleeve gastrectomy in the surgical treatment of morbid obesity have increased worldwide. Despite this, several aspects related to results at medium and long term remain in constant research.

**Aim:**

: To present the experience of sleeve gastrectomy in a center of excellence in bariatric surgery by analyzing clinical outcomes, complications and follow-up in the medium term.

**Methods:**

: The study included 120 morbidly obese patients who underwent sleeve gastrectomy and who were followed for at least 24 months. Aspects related to surgical technique, surgical complications and clinical outcome were analyzed.

**Results:**

: Seventy-five patients were women (62.5%) and the average age was 36 years. The body mass index preoperatively ranged from 35.5 to 58 kg/m^2^(average of 40.2 kg/m^2^). The length of stay ranged from 1 to 4 days (mean 2.1 days). Comorbidities observed were hypertension (19%), type 2 diabetes mellitus (6.6%), dyslipidemia (7.5%), sleep apnea (16.6%), reflux esophagitis (10%) and orthopedic diseases (7.5%). The mean body mass index and total weight loss percentage with 3, 12, 18 and 24 months were 32.2 kg/m^2^-19,9%; 29.5 kg/m^2^-26,5%; 28.2 kg/m^2^-30,3% and 26.9 kg/m^2^-32,7%, respectively. Remission of diabetes and dyslipidemia occurred in all patients. In relation to hypertension, there was improvement or remission in 86%. There were only two complications (bronchial pneumonia and dehydration), with good response to clinical treatment. There was no evidence digestive fistula and mortality was zero. Eleven patients (9.1%) had regained weighing more than 5 kg.

**Conclusion:**

: The sleeve gastrectomy is surgical technique that has proven safe and effective in the surgical treatment of obesity and control of their comorbidities in postoperative follow-up for two years.

## Abstract (pt)

**Racional:**

: As indicações da gastrectomia vertical no tratamento cirúrgico da obesidade mórbida têm aumentado no mundo todo. Apesar deste aumento, diversos aspectos relativos às indicações e aos resultados à médio e longo prazo permanecem em constante pesquisa.

**Objetivo:**

: Apresentar a experiência da gastrectomia vertical de um centro de excelência em cirurgia bariátrica, analisando os resultados clínicos, complicações e seguimento a médio prazo.

**Métodos:**

: Foram incluídos 120 pacientes obesos mórbidos submetidos à gastrectomia vertical e que foram acompanhados por no mínimo 24 meses. Foram analisados os aspectos relativos à técnica operatória, complicações cirúrgicas e evolução clínica.

**Resultados:**

: Dos pacientes estudados 75 eram mulheres (62,5%) e a idade média foi de 36 anos. O índice de massa corpórea pré-operatório variou de 35,5 a 58 Kg/m^2^ (média de 40,2 kg/m^2^). O tempo de internação variou de 1 a 4 dias (média de 2,1 dias). As co-morbidades observadas foram a hipertensão arterial sistêmica (19%), diabete melito tipo 2 (6,6%), dislipidemia (7,5%), apneia do sono (16,6%), esofagite de refluxo (10%) e doenças ortopédicas (7,5%). A média do índice de massa corpórea e do percentual de peso total perdido com 3, 12, 18 e 24 meses foram 32,2 kg/m^2^-19,9%; 29,5 kg/m^2^-26,5%; 28,2 kg/m^2^-30,3% e 26,9 kg/m^2^-32,7%, respectivamente. Houve remissão do diabete e da dislipidemia em todos os pacientes. Já em relação à hipertensão arterial sistêmica, houve melhora ou remissão em 86% dos casos. Ocorreram apenas duas complicações (broncopneumonia e desidratação), com boa resposta ao tratamento clínico. Não se evidenciou fístula digestiva e a mortalidade foi zero. Onze pacientes (9,1%) apresentaram reganho de peso superior a 5 kg.

**Conclusão:**

: A gastrectomia vertical é técnica operatória que se mostrou segura e eficaz no tratamento cirúrgico da obesidade e controle de suas co-morbidades em seguimento pós-operatório por dois anos.

## INTRODUCTION

From the definition of morbid obesity as a disease in the early 50s and the recognition of the high failure rates of conservative treatment with multidisciplinary monitoring, lifestyle changes and medication use, various surgical techniques have been proposed aimed at improvement of results in weight loss, co-morbidities control, decrease in complications and mortality. In this scenario, the sleeve gastrectomy (GV) is the latest proposition of bariatric surgery and also the fastest managed worldwide acceptance both by surgeons as by patients.

In recent years, there was an exponential increase in indicating the GV worldwide. Data obtained through electronic questionnaire showed that in 2003 it practically did not exist in the list of bariatric procedures performed in different regions of the world^10^ and in just 10 years, with progressive increase^11^^,^ 12, its indication grew-up to one third of world indications, and in the United States has already surpassed the percentage of Roux-en-Y gastric bypass^2^. The prospects are that these numbers continue to grow in coming years.

The possible reasons that can justify this growth may be related to the relative technical simplicity compared to other bariatric procedures, proper weight loss and good quality of life after the operation, especially without serious nutritional disorders in the long run. Furthermore, in the event of unsatisfactory results GV offers the possibility of revisional surgery using less complicated procedures in comparison to other bariatric surgeries.

Thus, despite the GV being very common bariatric procedure, also entails studies aimed to clarify controversial aspects regarding the surgical technique and to examine various aspects of the results, especially in long-term.

The aim of this study was to present the experience in GV of one excellence center in bariatric surgery, analyzing the technical aspects, complications and results after two years of follow-up.

## MÉTODOS

The study included all patients undergoing GV from July 2012 through June 2013 with follow-up until July 2015; so, it was possible to collected information until the 24^th^ month following the operation date. All had a diagnosis of morbid obesity with indication criteria for surgery with body mass index (BMI) greater than 40 or BMI greater than 35 with comorbidities. In the period selected for the study, it was not recommended to perform the GV in patients with type 2 diabetes mellitus (T2DM) with more than five years of history or using insulin; dyslipidemia; total cholesterol greater than 250 mg%; LDL over 150 mg% or triglycerides above 250 mg%; and gastroesophageal reflux disease (GERD) in upper grades of Los Angeles classification or hiatal hernia with size greater than 2 cm. Patients received, reviewed and signed the free and informed consent. All had multidisciplinary evaluation and follow-up with the surgeon, endocrinologist, cardiologist, nutritionist, psychologist and physiotherapist in the preparation period for the surgery ranged from 3-12 months and during the postoperative follow-up.

Technical systematization, patient preparation, positioning of the trocars, operative technique and postoperative care are presented in detail in another article in this same ABCD number ABCD 2015;28(Supl.1):65-68). Were evaluated in this article epidemiological data series; weight and BMI; comorbidities; length of stay; the most frequent reason for hospital stays longer than two days; re-admissions; the total percentage weight lost with 3, 12, 18 and 24 months; regained weighing more than 5 kg; operative complications; remission of T2DM, dyslipidemia and other comorbidities and orthopedic problems.

## RESULTS

**FIGURE 1:**
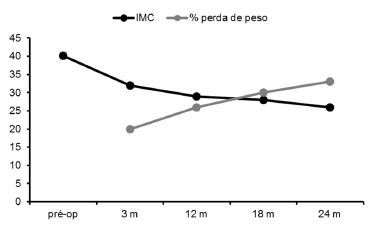
- Average reduction in BMI and increased weight loss percentage over the 24 months of postoperative observation of 120 patients undergoing laparoscopic GV

During the 12 months of the study were included 120 patients undergoing GV with the same technique; women represented 62.5% (n=75). The age ranged from 16-74 years (mean 36.2). Weight and BMI ranged from 82-175 kg (average 112.5) and 35.5-58 kg/m^2^ (mean 40.2), respectively. As comorbidities, 19% had hypertension, 6.6% T2DM, 7.5% dyslipidemia, 16.6% sleep apnea, 10% reflux esophagitis and 7.5% were suffering from orthopedic comorbidities, such as arthrosis and herniated disc. Some patients had more than one comorbidity. The average length of stay ranged from 1-4 days (mean 2.1). The most frequent reason for longer than two days hospital stay was the occurrence of nausea and vomiting in six patients (5%). Two were re-hospitalized, one due to bronchopneumonia (0.8%) and the other by dehydration (0.8%). The mean BMI and percentage of total weight lost with 3, 12, 18 and 24 months were 32,2 kg/m^2^-19,9%; 29,5 kg/m^2^-26,5%; 28,2 kg/m^2^-30,3% e 26,9 kg/m^2^-32,7%, respectively (Figure 1). Between the 18^th^ and 24^th^ months, 11 cases (9.1%) had regained weighing more than 5 kg. During the follow-up period, 12 (10%) were diagnosed with cholelithiasis, and none developed acute cholecystitis and all were undergoing elective laparoscopic cholecystectomy, uneventful. Complaints related to GERD were identified in 22 cases (18.3%), and all showed improvement with clinical treatment based on proton pump inhibitor and only 10 (8.3%) followed with continued use of the medication at the end of the study. Remission of T2DM (glycated hemoglobin below 6.5%) and dyslipidemia occurred in all patients. Regarding hypertension, 64.3% of patients had normalized their blood pressure (below 140/90 mmHg) without medication, 21.7% had reduced its need and 14% showed no change from the initial and continued with the same antihypertensive medication. All reported improvement in symptoms of their orthopedic problems. Among those with sleep apnea, 80% reported remission and 20% partial improvement.

## DISCUSSION

Since the first operations to treat morbid obesity in the 50's, bariatric surgeons produced several technical proposals in search of what could be considered the ideal method^8^.

With the advancement in understanding the pathophysiology of obesity and operating mechanisms of the various techniques of bariatric surgery, were added to the classical concepts of gastric restriction and intestinal malabsorption, some more modern aspects, such as metabolic disorders, neuro-hormonal alterations, incretinic effect, changes in the microbiota, absorption of bile acids, among others.

Among the accepted and globally practiced bariatric techniques, the Roux-en-Y gastric bypass is the most known for its risk-benefit^20^; but the biliopancreatic diversion with duodenal switch is considered the therapy that provides the best results both in weight loss aspect as in control of metabolic diseases associated with obesity, such as T2DM and dislipidemia^9^.

Thus, based upon the perspective of good long-term results, biliopancreatic diversion with duodenal switch has the classical indication for patients with the most severe form of morbid obesity: the super-obesity. The combination of higher severity and risk on bariatric surgery with greater size and complexity of the operation, invariably results in unacceptable rates of complications and mortality. 

In order to decrease the rate of complications was proposed the realization of the riskiest procedures in two stages. Initially, the GV would be held as it is a restrictive procedure with lower surgical time and easier technique, and, after a period of about a year being the patient already less obese, the operation would be completed with the intestinal derivation^23^. Although this operation in two stages is still held^17^, many centers have observed that patients submitted to GV chose not to perform the second procedure, once they were satisfied with the results of GV alone. These observations led to the proposition of GV isolated as a surgical technique for the surgical treatment of morbid obesity; nowadays, it has both indications: as unique procedure or as the first operative step in high risk patients^3^^,^ 7.

Moreover, some indications for GV still are controversial, such as the presence of GERD and obese with advanced metabolic syndrome. In this series, although GERD, T2DM and dyslipidemia were not considered as absolute contraindications, patients with more advanced stages of these diseases were not included, a fact that may have contributed to the good results observed for resolution or metabolic improvement, with complete remission of T2DM and dyslipidemia, in addition to the small number of patients with mild symptoms of gastroesophageal reflux at the end of two years.

In theory, smaller remnant stomach caliber could provide greater weight loss, at least in the initial postoperative period. However, this higher setting can also cause greater feeding difficulty and compromise the quality of life, and generate increased intragastric pressure with higher fistula risk in the suture line^27^. Another complication related directly to the reduction of stomach size is the narrowing or stenosis, with consequent gastric stasis, which can lead to recurrent episodes of vomiting, affecting the quality of life and increasing the risk of nutritional disorders.

The authors have been using lower caliber probe (32 Fr=10.7 mm) positioned next to the lesser gastric curvature, clipping next the edge of the probe with the intention of providing better weight loss in early postoperative. To allow better resection of the antrum, the probe is not used for the first shot. 

This adjustment of the stapling line to a probe of smaller caliber is controversial. There are studies that corroborate with this concept, confirming better weight loss, even in the long-term^4^. However, comparative studies with probes in different sizes have not shown differences in the results of weight loss in the first year^18^^,^ 26, and larger caliber tubes may even decrease the incidence of fistulas^19^ probably for allowing to perform the gastric tube with minor gastric luminal pressure.

Digestive fistulas are longtime feared complications for surgeons and patients. The main cause of these failures are in gastrointestinal anastomoses. Mechanical staplers are devices currently used because they allow gastrointestinal anastomoses more quickly done, especially in laparoscopy. In GV, fistula in staple line is the main complication in the immediate postoperative period, since it is most commonly detected within the first 10 days after the operation^22^. They occur in about 2% of patients undergoing GV^19^^,^ 24 and preferably located in the upper third of the gastric tube, especially near the esophagogastric transition^5^. In addition, the fistula due to GV is generally associated with higher morbidity, requiring the surgeon's knowledge of the various therapeutic modalities available such as percutaneous drainage^14^, endoscopic therapy with clips, dilation and stents^1^^,^ 25, simple suture of fistulous hole^15^, anastomosis between the fistula hole and jejunal loop^6^^,^ 13 and, more radically and reserved for selected cases and experienced surgeons, total gastrectomy with esophagojejunoanastomoses in Roux-en-Y^21^.

Although the average reported incidence of fistulas in the biomedical literature is about 2%, no leaks were observed in this series. The explanation may be related to the fact of the surgical team has started the regular practice of GV since 2005, providing better technical training during the study period.

In this series it was performed a continuous suture in a single layer with absorbable thread, not with the aim to prevent the occurrence of leaks, but to reduce the risk of postoperative bleeding at the staple line, both on its outer and inner surface, which could lead to episodes of upper gastrointestinal bleeding.

Resection of the antrum is another point of disagreement among surgeons, and some studies have shown that the more antrum is cut faster would be the gastric emptying, with consequent acceleration of intestinal transit and better metabolic effect, due to increased production of PYY and GLP 1, being this finding not coincident with other publications. On the other hand, studies evaluating the weight loss after GV correlated it to operation failure or failure of weight regained with the dilation or not adequate resection of fundus and antrum^16^.

In this analysis, all the gastric fundus was resected for wide release of the diaphragmatic crura and the antrum was resected from 2 to 3 cm of the pylorus. These three technical features - fundus resection, antrum ressection and calibration probe of 32 Fr - possibly played an important role in the final outcome as satisfactory overall weight loss. 

Based on the good results obtained with this series, as well as the low rate of complications, it seems that GV is effective technique feasible and safe for the surgical treatment of morbidly obese patients, since their technical steps are followed correctly. More studies are needed to get complete elucidation of several controversial points in order to consolidation GV as a definitive option for surgical treatment of obesity. 

## CONCLUSION

The sleeve gastrectomy is surgical technique that has proven to be safe and effective in the surgical treatment of obesity and in control of their comorbidities after follow-up period of two years.

## References

[1] Aly A, Lim HK (2013). The Use of Over the Scope Clip (OTSC) Device for Sleeve Gastrectomy
Leak. J Gastrointest Surg.

[2] Angrisani L, Santonicola A, Iovino P, Formisano G, Buchwald H, Scopinaro N (2015). Bariatric Surgery Worldwide 2013. Obes Surg..

[3] ASMBS Clinical Issues Committee (2012). Updated position statement on sleeve gastrectomy as a bariatric
procedure. Surg Obes Relat Dis..

[4] Atkins ER, Preen DB, Jarman C, Cohen LD (2012). Improved obesity reduction and co-morbidity resolution in patients
treated with 40-French bougie versus 50-French bougie four years after
laparoscopic sleeve gastrectomy. Analysis of 294 patients. Obes Surg..

[5] Aurora AR, Khaitan L, Saber AA (2012). Sleeve gastrectomy and the risk of leak: a systematic analysis of
4,888 patients. Surg Endosc.

[6] Baltasar A, Serra C, Bengochea M, Bou R, Andreo L (2008). Use of Roux limb as remedial surgery for sleeve gastrectomy
fistulas. Surg Obes Relat Dis.

[7] Brethauer SA, Hammel JP, Schauer PR (2009). Systematic review of sleeve gastrectomy as staging and primary
bariatric procedure. Surg Obes Relat Dis.

[8] Buchwald H, Buchwald JN (2002). Evolution of operative procedures for the management of morbid obesity
1950-2000. Obes Surg..

[9] Buchwald H, Avidor Y, Braunwald E, Jensen MD, Pories W, Fahrbach K, Schoelles K (2004). Bariatric surgery: a systematic review and
meta-analysis. JAMA..

[10] Buchwald H, Williams SE (2004). Bariatric Surgery Worldwide 2003. Obes Surg..

[11] Buchwald H, Oien DM (2009). Metabolic/Bariatric Surgery Worldwide 2008. Obes Surg..

[12] Buchwald H, Oien DM (2013). Metabolic/bariatric surgery worldwide 2011. Obes Surg..

[13] Chour M, Alami RS, Sleilaty F, Wakim R (2014). The early use of Roux limb as surgical treatment for proximal post
sleeve gastrectomy leaks. Surg Obes Relat Dis.

[14] Corona M, Zini C, Allegritti M, Boatta E, Lucatelli P, Cannavale A (2013). Minimally invasive treatment of gastric leak after sleeve
gastrectomy. Radiol Med.

[15] Csendes A, Braghetto I, León P, Burgos AM (2010). Management of Leaks After Laparoscopic Sleeve Gastrectomy in Patients
with Obesity. J Gastrointest Surg.

[16] Melissas J, Koukouraki S, Askoxylakis J, Stathaki M, Daskalakis M, Perisinakis K, Karkavitsas N (2007). Sleeve gastrectomy: a restrictive procedure?. Obes Surg..

[17] Mukherjee S, Devalia K, Rahman MG, Mannur KR (2012). Sleeve gastrectomy as a bridge to a second bariatric procedure in
super obese patients - a single institution experience. Surg Obes Relat Dis..

[18] Parikh M, Gagner M, Heacock L, Strain G, Dakin G, Pomp A (2008). Laparoscopic sleeve gastrectomy: does bougie size affect mean %EWL?
Short-term outcomes. Surg Obes Relat Dis..

[19] Parikh M, Issa R, McCrillis A, Saunders JK, Ude-Welcome A, Gagner M (2013). Surgical strategies that may decrease leak after laparoscopic sleeve
gastrectomy: a systematic review and meta-analysis of 9991 cases. Ann Surg..

[20] Ramos AC, Silva AC, Ramos MG, Canseco EG, Galvão-Neto Mdos P, Menezes Mde A, Galvão TD, Bastos EL (2014). Simplified gastric bypass: 13 years of experience and 12,000 patients
operated. Arq Bras Cir Dig..

[21] Ramos AC, Ramos MG, Campos JM, Galvão MP, Bastos EL (2015). Laparoscopic total gastrectomy as an alternative treatment to
postsleeve chronic fistula. Surg Obes Relat Dis..

[22] Rebibo L, Bartoli E, Dhahri A, Cosse C, Robert B, Brazier F, Pequignot A, Hakim S, Yzet T, Delcenserie R, Dupont H, Regimbeau JM (2015). Persistent gastric fistula after sleeve gastrectomy: an analysis of
the time between discovery and reoperation. Surg Obes Relat Dis..

[23] Regan JP, Inabnet WB, Gagner M, Pomp A (2003). Early experience with two-stage laparoscopic Roux-en-Y gastric bypass
as an alternative in the super-superobese patient. Obes Surg..

[24] Silva LB, Moon RC, Teixeira AF, Jawad MA, Ferraz ÁA, G M, Ramos AC, Campos JM (2015). Gastrobronchial Fistula in Sleeve Gastrectomy and Roux-en-Y Gastric
Bypass-A Systematic Review. Obes Surg..

[25] Slim R, Smayra T, Noun R (2013). Biliary endoprosthesis in the management of gastric leak after sleeve
gastrectomy. Surg Obes Relat Dis.

[26] Spivak H, Rubin M, Sadot E, Pollak E, Feygin A, Goitein D (2014). Laparoscopic sleeve gastrectomy using 42-French versus 32-French
bougie: the first-year outcome. Obes Surg..

[27] Yuval JB, Mintz Y, Cohen MJ, Rivkind AL, Elazary R (2013). The effects of bougie caliber on leaks and excess weight loss
following laparoscopic sleeve gastrectomy. Is there an ideal bougie
size?. Obes Surg..

